# Are All Domains of Life Satisfaction Equal? Differential Associations With Health and Well-Being in Older Adults

**DOI:** 10.1093/geroni/igab046.975

**Published:** 2021-12-17

**Authors:** Julia Nakamura, Scott Delaney, Ed Diener, Tyler VanderWeele, Eric Kim

**Affiliations:** 1 University of British Columbia, Vancouver, British Columbia, Canada; 2 Harvard T. H. Chan School of Public Health, Boston, Massachusetts, United States; 3 University of Virginia, Charlottesville, Virginia, United States; 4 Harvard T.H. Chan School of Public Health, Boston, Massachusetts, United States

## Abstract

Growing evidence documents strong associations between overall life satisfaction and favorable health and well-being outcomes. However, because most previous studies have assessed satisfaction with one’s life as a whole, we know little about whether specific domains of life satisfaction (e.g., satisfaction with income) might be driving better health and well-being outcomes. Data were from 13,752 participants in the Health and Retirement Study—a nationally representative cohort of US adults aged >or=50. We evaluated if positive changes in seven domains of life satisfaction (between t0;2008/2010 and t1;2012/2014) were associated with 35 indicators of physical, behavioral, and psychosocial health and well-being (at t2;2016/2018). Satisfaction with family life and non-work activities showed the largest associations with subsequent psychological factors, followed by satisfaction with financial situation and income. Effect estimates were double in magnitude for certain domains of life satisfaction (e.g., the association between satisfaction with family life and purpose in life (β=0.22, 95% CI:0.16,0.27) was more than twice as large as the association between satisfaction with housing and purpose in life (β=0.09, 95% CI:0.02,0.16). Further, some domains showed associations with physical health outcomes (e.g., participants with the highest satisfaction with health had a 21% decreased mortality risk (95% CI: 0.66,0.95)), health behaviors (e.g., higher satisfaction with income decreased risk of sleep problems by 11% (95% CI:0.80,0.99)), and social factors (e.g., loneliness (β: -0.16 to -0.42)). Individual domains of life satisfaction might be novel targets for interventions and policies seeking to enhance specific facets of health and well-being in our rapidly aging population.

